# Preparation and characterization of soluble dietary fiber from tiger nut residues, showing enhanced antioxidant activity and metal-ion-binding properties

**DOI:** 10.3389/fnut.2023.1275473

**Published:** 2023-12-12

**Authors:** Weihao Wang, Zhigang Quan, Fang Kou, Shenglong Zhang, Longkui Cao, Zhi Zhang

**Affiliations:** ^1^School of Forestry, Northeast Forestry University, Harbin, China; ^2^National Coarse Cereals Engineering Research Center, Heilongjiang Bayi Agricultural University, Daqing, China; ^3^College of Food Science, Heilongjiang Bayi Agricultural University, Daqing, China; ^4^Department of Marine Food Science and Technology, Gangneung-Wonju National University, Gangneung, Republic of Korea; ^5^Heilongjiang Guohong Energy Saving and Environmental Protection Co., Harbin, China; ^6^School of Life Science, Northeast Forestry University, Harbin, China

**Keywords:** superfine grinding, soluble dietary fiber, preparation, structure, activity

## Abstract

To improve the utilization of soluble dietary fiber (SDF) from tiger nut residues, the response surface methodology was used to optimize the conditions of superfine grinding to produce SDF with antioxidant and metal-ion-binding properties. The yield was increased (30.56%) and the average particle diameter of SDF was decreased (D50: 32.80 μm) under the optimal conditions (a proportion of grinding medium of 100%, a feeding mass of 0.90 kg, a grinding time of 20 min, and a moisture content of 8.00%). In addition, superfine grinding substantially modified the surface morphology and increased the SDF content and the proportion of monosaccharides by decreasing the molecular weight. Moreover, superfine grinding remarkably enhanced the *in vitro* antioxidant activities (ABTS^+^, DPPḤ, and ·OH) of the SDF, which also exhibited favorable metal-ion-binding properties (Ca^2+^, Zn^2+^, and Co^2+^). These results suggest that superfine grinding can be used as a technique to modify dietary fiber to manufacture functional SDF.

## 1 Introduction

Tiger nuts (*Cyperus esculentus L.*) are some of the lesser-known edible perennial vegetables of the tuber of *Cyperus esculentus L*. They are widely cultivated in Spain, Burkina Faso, Mali, Niger, and Nigeria and are used as a food ingredient and feed (1). Tiger nuts are widely praised for their health benefits and nutritional value as they have high levels of protein, oleic acid, vitamins, polyphenols, and dietary fiber (59.71 g/100 g) (2). The dietary fiber from tiger nut by-products can help prevent heart disease and thrombosis, and reduce the risk of diabetes and colon cancer (2).

Numerous studies have shown that dietary fiber exerts substantial effects in promoting intestinal health, regulating intestinal microorganisms, protecting the colonic mucosa, enhancing immunity, and improving glucose and lipid metabolism (3–5). However, the primary contributor to these health benefits is soluble dietary fiber (SDF), which can be used by gut microorganisms to produce short-chain fatty acids and other metabolites (6). These metabolites participate in various metabolic pathways that promote host health (6). On the other hand, water-insoluble dietary fiber (IDF) is not absorbed by the body or utilized by intestinal microorganisms but promotes intestinal motility and helps prevent diverticular disease and colon cancer. Therefore, increasing the content of SDF is crucial for the preparation of fiber-rich fortified foods with health benefits. Unfortunately, these nutrients—particularly SDF—cannot be efficiently utilized because they are usually discarded together with by-products, which leads to a serious wastage of resources and environmental pollution (7). Thus, the development of SDF-rich foods using tiger nut by-products is an attractive strategy for overcoming nutrient losses and avoiding resource waste.

Superfine grinding is an environmentally friendly processing technology with excellent micro-powder modification properties. This technology not only effectively alters the structure of dietary fibers by redistributing them from the insoluble to the soluble fractions (8), but also exposes more glucuronic/galacturonic acid and ionic binding sites in the modified fiber molecules (9). For instance, with the exposure of ionic sites, some essential micronutrients such as Co^2+^, Zn^2+^, Fe^2+^, and Ca^2+^ are adsorbed to the modified dietary fiber, which not only improves the bioavailability of metal ions but also alleviates the occurrence of diseases caused by micronutrient deficiencies (10). Moreover, these structural changes also improve the physicochemical properties and processing performance of dietary fibers and the antioxidant activity of modified dietary fibers (11). Therefore, superfine grinding technology plays an important role in the development of functionally modified dietary fibers.

Unfortunately, although research has shown that superfine grinding technology exhibits excellent micro-powder modification properties, its effect on the modification of dietary fibers from tiger nuts as well as the antioxidant capacity of the modified SDF and the exchange of metal ions has not been reported, limiting the comprehensive utilization pathway of byproducts from tiger nuts. To investigate the application of superfine grinding technology in modifying the antioxidant and ionic binding capacity of dietary fibers and increasing the content of SDF, we optimized the superfine grinding process by response surface methodology and comparatively analyzed the effects of superfine grinding on the structural properties, antioxidant activity, and metal-ion-binding properties of SDF. The results of this study are expected to demonstrate the feasibility of superfine grinding in modifying dietary fibers and developing functional fiber-rich nutritional supplements.

## 2 Materials and methods

### 2.1 Materials

Tiger nut residues were obtained from the National Coarse Cereals Engineering Research Center of Heilongjiang Bayi Agricultural University (Daqing, China). α-Thermostable amylase (40,000 U/mL), neutral protease (60,000 U/mL), amyloglucosidase (100,000 U/mL), 1,1-diphenyl-2-picrylhydrazyl (DPPḤ), 2,2′-azinobis-(3-ethylbenzthiazoline-6-sulfonate; ABTS^+^), and 2,4,6-tripyridin-2-yl − 1,3,5-triazine (TPTZ) and monosaccharide standards (Mannose, Ribose, Rhamnose, Glucose, Galactose, Xylose, Arabinose, Fucose, Glucuronic acid, Galacturonic acid) were acquired from Sigma (St. Louis, MO, United States). All chemicals and solvents were of analytical grade.

### 2.2 Preparation of SDF

#### 2.2.1 Superfine grinding

The defatted tiger nut residues were ground using a high-speed crusher (FW80, Taisete Co., Ltd., Tianjin, China) and the particles were collected after passing them through a 60-mesh sieve. A JFM-50 L vibrating superfine mill (Jianchen Machinery Co., Ltd., Jinan, China) was used to prepare the superfine powder. The parameters of the process according to the experimental design were as follows: moisture contents of 4, 6, 8, 10, and 12%; feeding mass of 0.25, 0.5, 0.75, 1.00, and 1.25 kg; grinding times of 5, 10, 15, 20, and 25 min; and grinding medium proportions of 60, 70, 80, 90, and 100%.

The particle size distribution of the superfine powder was measured using a Bettersize 2000 laser particle size analyzer (Better Instrument Co., Ltd., Dandong, China). The D50 was used to characterize the median particle diameter of the superfine powder. The contents of soluble dietary fiber (SDF), insoluble dietary fiber (IDF), and total dietary fiber (TDF) in the pretreated samples were determined using the AOAC method 991.43 (AOAC, 1996).

#### 2.2.2 Extraction of soluble dietary fiber from tiger nut residues

SDF was extracted by applying an enzyme-assisted method according to the principles of the AOAC Official Methods 985.29 and 960.52 (12), with modifications. Briefly, defatted tiger nut residue powder was mixed with deionized water (1:50, w/v), and appropriate amounts of α-water-repellent amylase, neutral protease, and amyloglucosidase were added sequentially. The mixture was inactivated by enzymatic digestion in a GDE Enzymatic Digester (VELP Scientifica, Usmate, Italy). Subsequently, the supernatant was collected by centrifugation at 4000 rpm for 20 min and concentrated to one-fourth of the original volume. The precipitate was collected by sedimentation in 95% ethanol (1,4, v/v) for 12 h at 4°C, and lyophilized at-108°C for 48 h to obtain crude SDF. SDF (M1) obtained after superfine grinding (S-SDF) and ordinary crushing (O-SDF) was purified by removing the protein, using the Sevag method.


(1) $$SDF\;\mathrm{yield}\;\left(\%\right)=\frac{M1}{M2}\times 100\%$$

where M_1_ is the weight of the purified SDF (mg) and M_2_ is the weight of the defatted tiger nut residue powder (mg) used.

### 2.3 Experimental design and RSM optimization

To further investigate the relationship between the interaction of variables, we utilized response surface analysis to determine the optimal extraction process based on SDF yield and D50 indexes according to the Box–Behnken sampling principle and the initial screening results of the single factor. Herein, four independent variables (grinding time A, moisture content B, feeding mass C, and the proportion of the grinding medium D) were coded into three levels, and 29 experiments were conducted using a Box–Behnken design. The results are shown in Tables 1, 2.

**Table 1:** Response surface test factor levels and coding.

Coding	Grinding time A/(min)	Moisture content B/%	Feeding mass C/kg	Proportion of the grinding medium D/%
-1	15	4	0.50	80
0	20	6	0.75	90
1	25	8	1.00	100

**Table 2:** Results of the Box–Behnken experimental design.

Run	Grinding time (min)	Moisture content (%)	Feeding mass (kg)	Grinding medium proportion (%)	D50 value (μm)	SDF yield (%)
1	20.00	8.00	0.50	90.00	23.54	29.79
2	20.00	6.00	0.75	90.00	22.08	28.04
3	15.00	6.00	0.75	80.00	18.73	18.94
4	15.00	6.00	0.75	100.00	26.83	22.95
5	20.00	4.00	0.50	90.00	20.34	25.78
6	15.00	4.00	0.75	90.00	20.08	19.45
7	20.00	8.00	1.00	90.00	27.32	31.08
8	20.00	4.00	1.00	90.00	26.74	27.04
9	20.00	8.00	0.75	80.00	17.58	28.16
10	15.00	6.00	0.50	90.00	16.89	22.25
11	20.00	4.00	0.75	80.00	21.04	27.72
12	20.00	6.00	1.00	100.00	30.16	29.65
13	15.00	6.00	1.00	90.00	30.80	18.79
14	20.00	8.00	0.75	100.00	30.57	32.69
15	20.00	6.00	1.00	80.00	23.42	26.44
16	20.00	6.00	0.75	90.00	21.60	27.68
17	25.00	6.00	1.00	90.00	24.28	24.00
18	20.00	6.00	0.75	90.00	22.07	28.04
19	25.00	6.00	0.75	100.00	28.93	20.26
20	20.00	6.00	0.75	90.00	21.60	28.55
21	20.00	6.00	0.50	80.00	16.03	26.79
22	25.00	6.00	0.50	90.00	27.47	18.57
23	25.00	4.00	0.75	90.00	26.49	20.20
24	20.00	6.00	0.50	100.00	27.09	26.18
25	20.00	6.00	0.75	90.00	21.66	28.04
26	25.00	8.00	0.75	90.00	23.78	24.10
27	15.00	8.00	0.75	90.00	26.39	24.00
28	20.00	4.00	0.75	100.00	23.89	25.54
29	25.00	6.00	0.75	80.00	20.20	22.24

### 2.4 Structural characterization

#### 2.4.1 Ultraviolet–visible spectroscopy

The structural properties of the SDF samples were analyzed by Ultraviolet–Visible (UV–Vis) spectroscopy (Frontier, PerkinElmer, Shelton, CT, United States) in the range of 190–420 nm. SDF samples (1.5 mg/mL) were stained with phenol sulfuric acid, and the dyed samples were compared with a blank distilled water background.

#### 2.4.2 Scanning electron microscopy

Morphological and microstructural studies of the SDF samples were performed using scanning electron microscopy (SEM; SU8020, Hitachi, Ltd., Tokyo, Japan). The lyophilized SDF samples were mounted on a circular aluminum stub and sputter-coated with gold before the SEM observations. The images were collected at an accelerating voltage of 5.0 kV. Micrographs were recorded at 2000× magnification.

#### 2.4.3 Atomic force microscopy

The topographical properties of the SDF samples were determined by atomic force microscopy (AFM; Dimension Icon, Bruker AXS, Karlsruhe, Germany). In a typical procedure, a drop of properly diluted SDF (6 mg/mL) was placed on a mica slide and dried at room temperature before analysis in the dynamic mode (tapping mode).

#### 2.4.4 Monosaccharide composition

The monosaccharide composition of the SDF was analyzed by high-performance liquid chromatography (HPLC). Samples (5 ± 0.05 mg) were hydrolyzed with 2 M trifluoroacetic acid (TFA) in a sealed glass tube at 120°C for 5 h. After flushing with N_2_ to remove the remaining acid, the residue was dissolved in methanol (2 mL) subsequently flushed with N_2,_ and dried three times. The solution was analyzed by the Agilent 1,200 HPLC system (Agilent Technologies Co., Ltd., Santa Clara, CA, United States) with a flow rate of 0.5 mL/min, and injection volume is 5 μL. All measurements were conducted in triplicate. Mannose, ribose, rhamnose, glucose, galactose, xylose, arabinose, fucose, glucuronic acid, galacturonic acid were selected as standards for monosaccharides.

#### 2.4.5 Molecular weight

The molecular weights of the SDF samples were determined by gel permeation chromatography (GPC). SDF (60 ± 1.0 mg) was dissolved in 10 mL of a NaNO_3_ solution (0.1 mol/L). The chromatographic stationary phase was a porous gel, while the mobile phase was NaNO_3_ (0.1 mol/L). A TSKgel GMPWxl aqueous gel chromatographic column (H&E Co., Ltd., China) was used. The column temperature was 35°C and the loading volume was 20 μL.

#### 2.4.6 Fourier-transform infrared spectroscopy

Differences in the molecular structure of the SDF samples were identified by Fourier-transform infrared spectroscopy (Frontier, PerkinElmer, Shelton, CT, United States) in the range of 400 to 4,000 cm^−1^. SDF (2 ± 0.05 mg) was mixed with 200 mg of KBr, the mixture was ground and pressed into tablets. Scans were compared with a blank KBr background.

### 2.5 Cation-exchange capacity

The cation-exchange capacity was determined based on a published method (13), with slight modifications. Briefly, SDF samples (0.5 ± 0.01 g) were mixed with 30 mL of hydrochloric acid (0.01 mol/L) and the suspensions were stirred at 4°C for 24 h. The insoluble residue was washed with distilled water until the filtrate was free of Cl^−^ (verified with a 10% AgNO_3_ solution). The residue was then titrated with a NaOH solution (0.01 mol/L) using phenolphthalein as an indicator until the pH remained constant. Cation exchange capacity was expressed as meq OH^−^ per gram of dry samples. Hydrochloric acid (0.01 mol/L) without sample was used as control group.

### 2.6 Metal-ion-binding properties

The metal-ion-binding properties were evaluated based on previous studies (14, 15), with some modifications. Briefly, Zn(II), Ca(II), and Co(II) were added to the SDF solution (5 mg/mL) until the final concentration reached 0.01 mol/L. The initial pH values of the solutions were adjusted to 2–9 using different concentrations of HCl or NaOH at 50°C. Once the adsorption equilibrium was reached by shaking in a water bath, the supernatant was precipitated with 95% ethanol (1,4, v/v), and the precipitate was washed three times with 75% ethanol and lyophilized. The content of metal ions was determined using an atomic absorption spectrophotometer (AAS; Shimadzu, Tokyo, Japan). The quantity of metal ions bound to the SDF was calculated as follows:


(2) $$\mathrm{Quantity of metal}\;\mathrm{ion}\;\mathrm{bind}\;\left(\mathrm{mmol}/g\right)=\frac{CV/M}{\left(1-CV/M\right)Ar}\times 1000$$

where *C* is the concentration of metal ions determined by AAS (mg/mL), *V* is the sample volume (mL), *M* is the mass of the metal ion complex used for AAS determination (mg), and *Ar* is the relative atomic mass of the metal element.

### 2.7 Antioxidant capacity *in vitro*

#### 2.7.1 DPPH^•^radical-scavenging activity

The DPPH^•^ radical-scavenging activity assay was performed based on previous studies (16, 17), with some modifications. Briefly, an SDF solution (1–6 mg/mL) and a DPPḤ solution (0.1 mM) were mixed and incubated at 25°C for 30 min in the dark. For the assay, the decrease in absorbance (517 nm) was recorded after 30 min, and the percentage of free radical scavenging activity was determined from the difference in the absorbance between the control (vitamin C (V_C_)) and the samples. The radical-scavenging rate was calculated as follows:

(3) $$\mathrm{DPPH}\cdot \mathrm{scavenging}\;\mathrm{rate}\;\left(\%\right)=\left(1-\frac{A_{1}-A_{2}}{A_{0}}\right)\times 100\%$$

where A_1_, A_2_, and A_0_ are the absorbances of the SDF mixed with the DPPḤ solution, the SDF without the DPPḤ solution, and the DPPḤ solution without SDF, respectively.

#### 2.7.2 ABTS^+^ radical-scavenging activity

The ABTS^+^ radical-scavenging activity assay was conducted according to previous studies (16, 17), with some modifications. Briefly, an SDF solution (1–6 mg/mL) and an ABTS^+^ solution were mixed. For the assay, the decrease in absorbance (734 nm) was recorded after 30 min, and the percentage of free radical scavenging activity was determined from the difference in the absorbance between the control (vitamin C (V_C_)) and the samples. The radical-scavenging rate was calculated as follows:


(4) $${\mathrm{ABTS}}^{+}\;\mathrm{scavenging}\;\mathrm{rate}\;\left(\%\right)=\left(1-\frac{A_{1}}{A_{0}}\right)\times 100\%$$

where A_1_ and A_0_ are the absorbances of the ABTS^+^ solution with SDF and the ABTS^+^ solution without SDF, respectively.

#### 2.7.3 Hydroxyl radical-scavenging activity

The hydroxyl radical-scavenging activity assay was performed according to previous publications (16, 17), with some modifications. Briefly, an SDF solution (1–6 mg/mL) and a FeSO_4_ solution were mixed. The decrease in absorbance (510 nm) was recorded after 60 min, and the percentage of free radical scavenging activity was determined from the difference in the absorbance between the control (vitamin C (V_C_)) and the samples. The radical-scavenging rate was calculated as follows:


(5) $$\cdot OH\;\mathrm{scavenging}\;\mathrm{rate}\;\left(\%\right)=\left(1-\frac{A_{1}}{A_{0}}\right)\times 100\%$$

where A_1_ and A_0_ are the absorbances of the experimental group and the control group, respectively.

### 2.8 Statistical analysis

All experiments were performed in triplicate. The values were averaged and the standard deviation was calculated. SPSS 25.0 (IBM Corp., Armonk, NY, United States) was used to analyze the data. The final results were expressed as mean ± SD. *p* < 0.05 was regarded as significant.

## 3 Results and discussion

### 3.1 Single-factor experiments

To maximize the modification capacity of the soluble dietary fiber (SDF), we optimized the critical steps of superfine grinding including the grinding time, moisture content, feeding mass, and the proportion of the grinding medium.

**Figure 1:**
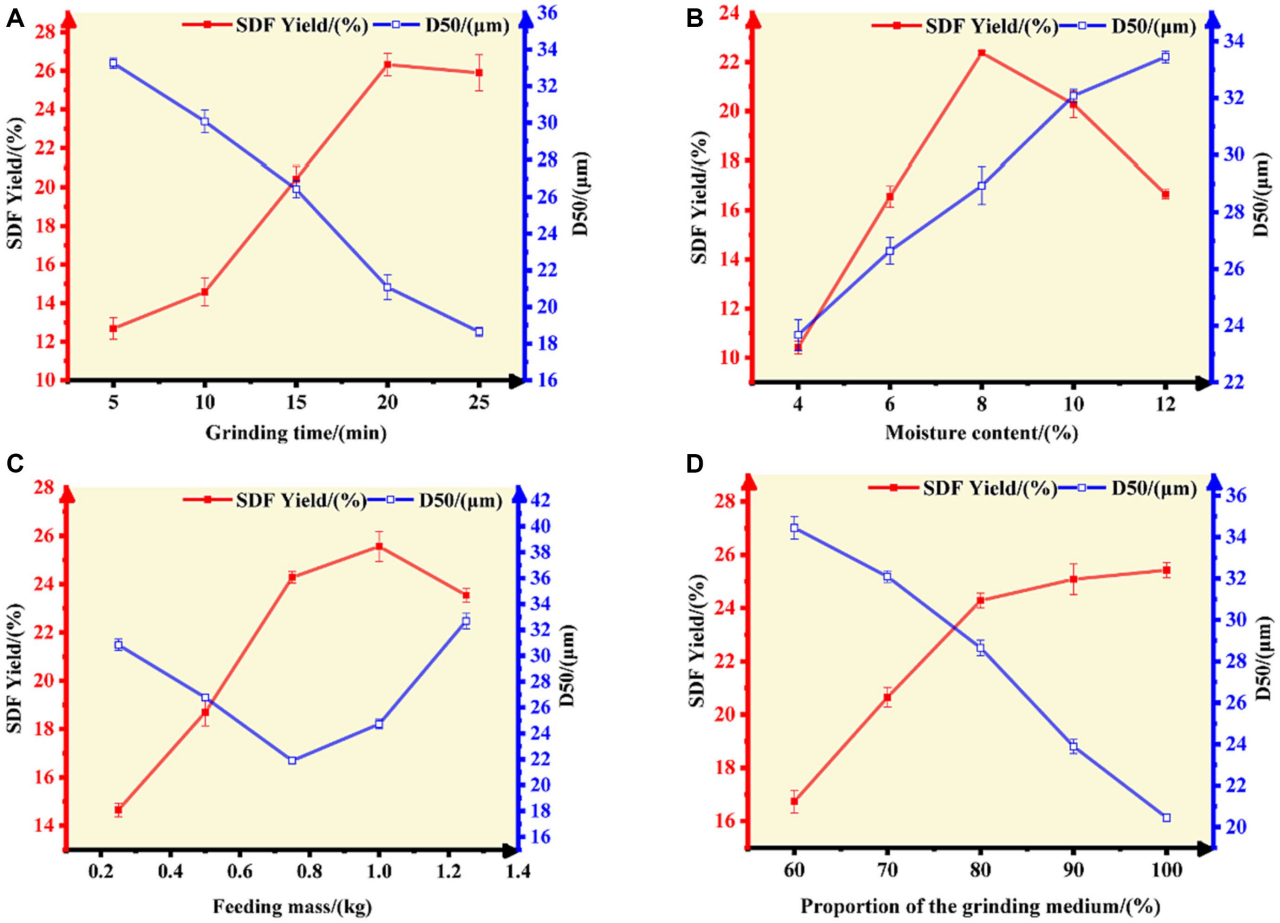
Effect of **(A)** grinding time, **(B)** moisture content, **(C)** feeding mass, and **(D)** the proportion of the grinding medium on the SDF yield and D50 value of tiger nut residues.

The grinding time and moisture content of the material determine the yield of SDF, which exhibited a trend of rapid increase (the peak value was obtained at 20 min of pulverization time, while the moisture content was 8.00%) followed by a minor decrease (Figures 1A,B). The increase in the content of SDF is attributed to the fact that superfine grinding (S-SDF) is more conducive to the destruction of the fibrous tissue structure, exposing the hydrophilic groups in the fiber’s cell walls with the accompanying reduction in particle size (the D50 reached 18.64 μm at 25 min; Figure 1A). This improves the solubility of the dietary fiber and facilitates the conversion of IDF to SDF. However, the excessive duration of superfine grinding and the high temperature generated inside the machine under high-speed vibration leads to the evaporation of free moisture from the material. This moisture accelerates the adhesion of the material to the inner wall of the machine, resulting in a decreasing SDF yield and increasing D50 (Figure 1B). Therefore, the grinding time and moisture content of the material are critical factors that affect the characteristics of the resulting SDF powder. The moisture content should be maintained within 8.00% and the grinding time should not be more than 20 min.

In addition to the grinding time and moisture content, the effects of the feeding mass and the proportion of the grinding medium on the SDF yield and D50 value were also remarkably. The SDF yield increased gradually with an increase in the feeding mass (0.20–1.00 kg). The yield decreased when the feeding mass exceeded 1.00 kg, in which case the D50 increased slightly (when the feeding mass exceeded 0.75 kg) because of insufficient grinding and shearing (Figure 1C). By contrast, an increase in the proportion of the grinding medium increased the SDF yield and was accompanied by a gradual decrease in particle diameter (D50; Figure 1D). This is due to the fact that the proper feeding mass and grinding medium proportion (0.20–1.00 kg; 80%) increases the frequency of collisions between the material and the medium, thus increasing the shear and friction forces inside the chamber of the superfine grinding equipment, which is more conducive to destroying the structure of the dietary fibers. These physical impacts, extrusion, and shear caused by the crushing of the material promote the conversion of IDF to SDF. On the other hand, when the feed volume is higher than a certain limit, the vibration space in the inner chamber of the equipment is reduced, and the material aggregates and has little mobility, resulting in reduced shear and friction between the fiber molecules, therefore limiting the pulverizing effect.

### 3.2 RSM optimization of extraction conditions

#### 3.2.1 Model fitting and statistical analysis

To further optimize the superfine grinding conditions to attain a high SDF yield and small particle diameter, we used the Design-Expert 10.0.3 software for the analysis of variance (grinding time, moisture content, feeding mass, and the proportion of the grinding medium) of the D50 value and SDF yield. The selection of these variables was based on the results of a single-factor experiment (moisture content of 8.00%, grinding time of less than 20 min, feed mass of 1.00 kg, and grinding media proportion of 80%). The results showed that the optimized quadratic equation for the D50 value was: D50 (μm) = 28.07 + 0.25 A + 2.01 B + 0.64 C + 0.58 D - 0.16 AB + 2.22 AC - 1.50 AD + 7.5 × 10^−3^ BC + 1.68 BD + 0.95 CD - 6.76 A^2^ + 0.72 B^2^ - 0.43 C^2^ - 0.28 D^2^.

Subsequently, we analyzed the regression equation of the D50 value by ANOVA, and the results are shown in Table 3. The *F*-value can be used to test the level of significance of each variable on the response value. The larger the F-value, the higher the level of significance of the corresponding variable. The model is considered statistically significant when *p* < 0.05. The effects of the processing conditions on D50 were in the order of B > C > D > A (i.e., moisture content > feeding mass > proportion of grinding medium > grinding time; Table 3). The coefficient of determination of the model (*R^2^* = 0.9979) indicates that the model is highly significant. Meanwhile, R^2^adj = 0.9958 (i.e., it can explain 99.58% of the experimental response variation) and is close to the predicted correlation coefficient (Pred R^2^), indicating that this experimental model fits well with the real data and is of practical significance. Hence, the model can be used to analyze and predict the optimal extraction process of SDF from tiger nut residues based on the D50 value. Based on the above, the optimized extraction process according to the Box–Behnken design to obtain the best particle diameter (D50) was 20.81 min of grinding time, a moisture content of 7.92%, a feeding mass of 0.92 kg, and a grinding medium proportion of 97.64%. The model predicted a D50 value of 32.78 μm under these conditions.

**Table 3:** Analysis of variance of the D50 value obtained with the Box–Behnken design.

Source^a^	Sum of squares	DF	Mean square	F-value	P-value prob. > F	Significant^b^
Model	429.34	14	30.67	474.46	<0.0001	**
A	0.75	1	0.75	11.53	0.0044	**
B	48.36	1	48.36	748.20	<0.0001	**
C	4.86	1	4.86	75.25	<0.0001	**
D	4.06	1	4.06	62.81	<0.0001	**
AB	0.11	1	0.11	1.63	0.2219	
AC	19.76	1	19.76	305.68	<0.0001	**
AD	8.97	1	8.97	138.78	<0.0001	**
BC	0.00025	1	0.00025	0.003481	0.9538	
BD	11.26	1	11.26	174.14	<0.0001	**
CD	3.65	1	3.65	56.44	<0.0001	**
A^2^	296.27	1	296.27	4583.66	<0.0001	**
B^2^	3.33	1	3.33	51.54	<0.0001	**
C^2^	1.21	1	1.21	18.74	0.0007	**
D^2^	0.52	1	0.52	7.99	0.0135	*
Residual	0.90	14	0.065			
Lack of fit	0.52	10	0.052	0.54	0.8054	
Pure error	0.39	4	0.096			
Cor total	430.24	28				
R^2^	0.9979					
Adj R^2^	0.9958					
Pred R^2^	0.9916					

^a^A, grinding time; B, moisture content; C, feeding mass; D, grinding medium proportion. ^b^*Significant, *P* < 0.05; **highly significant, *p* < 0.001.

Similarly, the optimal conditions for obtaining a high SDF yield were identified using the Design-Expert 10.0.3 software for analysis of variance. The resulting quadratic multinomial regression equation was: the yield of SDF (%) = 21.80 + 0.95 A + 0.88 B + 2.61 C + 4.21 D–2.26 AB–4.27 AC + 0.16 AD–0.65 BC + 2.54 BD–1.08 CD + 1.35 BD + 2.61 C + 4.21 D–2.26 AB - 4.27 AC + 0.16 AD–0.6 BC + 2.54 BD–1.08 CD + 1.35 A^2^ + 0.96 B^2^ + 1.75 C^2^ + 0.55 D^2^.

In addition, the influence of the superfine grinding conditions on the yield of SDF was in the order of D > C > A > B (i.e., grinding medium proportion > feeding mass > grinding time > moisture content; Table 4). Subsequently, the Box–Behnken design was used to optimize the extraction conditions. The model predicted an SDF yield of 32.10% when the grinding time was 24.88 min, the moisture was 5.08%, the feeding mass was 0.51 kg, and the grinding medium proportion was 99.26%.

**Table 4:** Analysis of variance of the SDF yield obtained with the Box–Behnken design.

Source^a^	Sum of squares	DF	Mean square	F-value	P-value prob. > F	Significant^b^
Model	468.48	14	33.46	519.62	<0.0001	**
A	10.89	1	10.89	169.06	<0.0001	**
B	9.36	1	9.36	145.40	<0.0001	**
C	81.95	1	81.95	1272.60	<0.0001	**
D	212.27	1	212.27	3296.14	<0.0001	**
AB	20.34	1	20.34	315.84	<0.0001	**
AC	73.10	1	73.10	1135.15	<0.0001	**
AD	0.099	1	0.099	1.54	0.2349	
BC	1.72	1	1.72	26.65	0.0001	*
BD	25.70	1	25.70	399.15	<0.0001	**
CD	4.67	1	4.67	72.45	<0.0001	**
A^2^	11.82	1	11.82	183.52	<0.0001	**
B^2^	5.99	1	5.99	93.04	<0.0001	**
C^2^	19.89	1	19.89	308.85	<0.0001	**
D^2^	1.96	1	1.96	30.45	<0.0001	**
Residual	0.90	14	0.064			
Lack of Fit	0.65	10	0.065	1.04	0.5317	
Pure Error	0.25	4	0.063			
Cor Total	469.38	28				
R^2^	0.9981					
Adj.R^2^	0.9962					
Pred R^2^	0.9912					

^a^A, grinding time; B, moisture content; C, feeding mass; D, grinding medium proportion. ^b^*Significant, *P* < 0.05; **highly significant, *p* < 0.001.

#### 3.2.2 Response surface analysis

**Figure 2:**
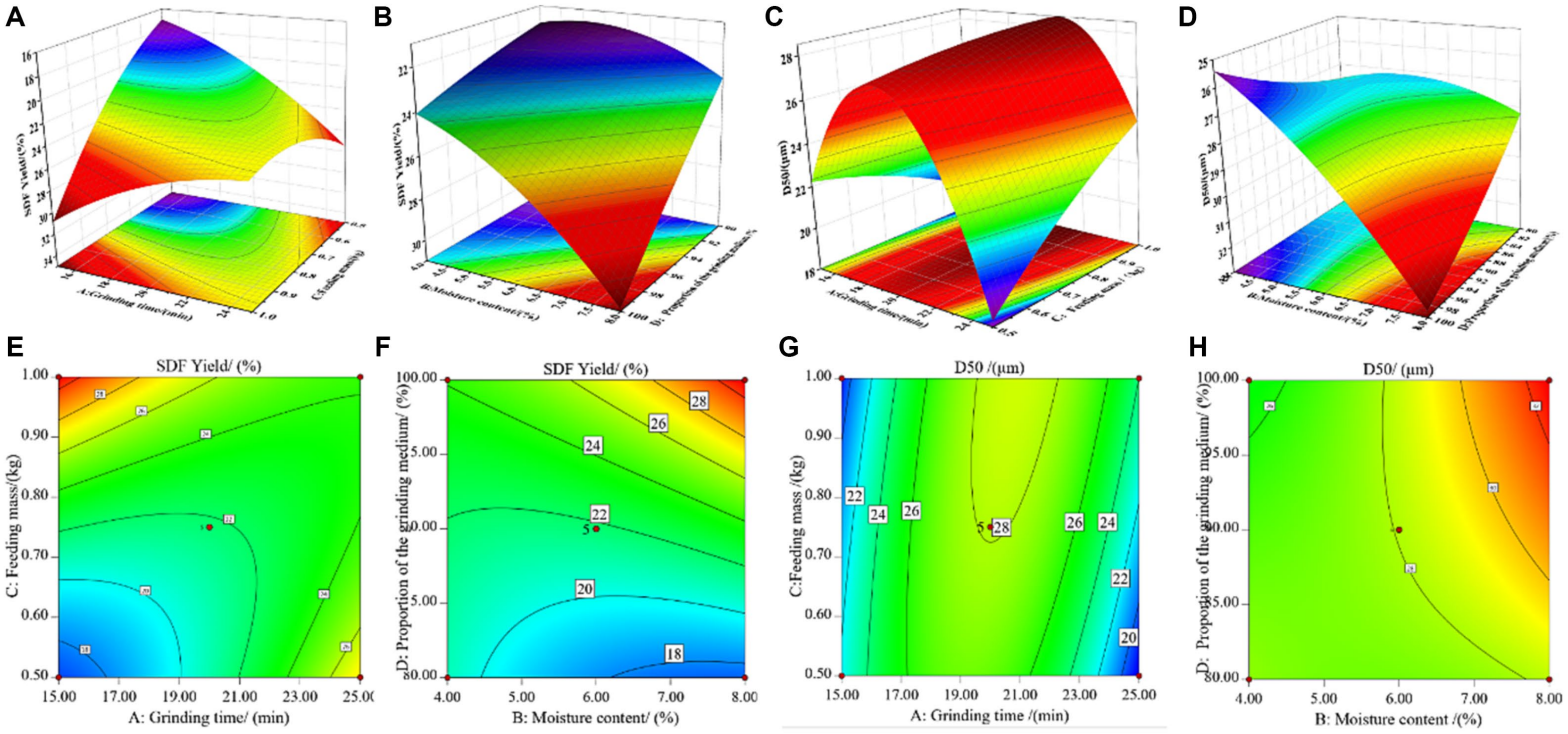
Response surface plots **(A–H)** showing the effects of the most substantial interaction variables [grinding time (min) and feeding mass (kg), moisture content (%) and the proportion of the grinding medium (%)] on the SDF yield and D50 value of tiger nut residues.

By analyzing the ANOVA results presented in Tables 3, 4, we have observed that a higher degree of significance is linked to interaction variables when the *p*-value falls below 0.0001, and their corresponding *F*-values are notably larger. Hence, we have chosen the most substantial interaction variables, denoted as AC and BD, and have represented them graphically on 3D response surfaces and contour plots, as depicted in Figure 2. These graphical representations were employed to elucidate the impact of these variables and their interactions on both SDF yield and D50 values.

Figures 2A,E depict the mutual influence of grinding time and feed mass. The graphs exhibit a parabolic distribution, with a significant vertical span for grinding time, indicating its pronounced sensitivity to SDF yield within the interaction of these two factors. As shown in Figures 2B,F, the effects of moisture content and grinding medium proportion on SDF yield follow a trend of initially increasing and then decreasing. Under moderate conditions, specifically with moisture content between 5.0–7.0% and a grinding medium proportion of around 85–95%, it is conducive to enhancing SDF yield. According to the data in Figures 2C,G, the mutual influence of grinding time and feed mass exhibits a significant arch-shaped pattern, with grinding time still being a crucial influencing factor on SDF yield within their interaction. When considering only the impact of these two factors, the optimal process conditions for SDF yield should remain within a range of 19–21 min of grinding time. From Figures 2D,H, it can be observed that the variation in D50 is less pronounced due to changes in feed mass compared to the impact of grinding medium proportion. When the grinding medium proportion is below 90%, D50 correlates positively with it. However, when the grinding medium proportion exceeds 90%, their relationship undergoes a transformation, with the optimal process parameter being a grinding medium proportion of approximately 90%. Additionally, D50 increases initially and then levels off with an increase in moisture content.

According to the results of the software prediction and combined with the feasibility of the actual process setup, the optimal processing conditions were: a grinding time of 20 min, a moisture content of 8.00%, a feeding mass of 0.90 kg, and a grinding medium proportion of 100%. These conditions produced results that were close to the actual results from three replicated tests including the average D50 value (32.80 μm) and SDF yield (30.56%). Therefore, the optimization of the SDF extraction conditions based on this response surface model is feasible.

### 3.3 Analysis of components of the dietary fiber from tiger nut residues

SDF from tiger nut residues was extracted based on the optimized conditions for superfine grinding and its composition was analyzed. The contents of total dietary fiber (TDF) obtained after superfine grinding (S-SDF) and ordinary crushing (O-SDF) were similar. However, the contents of IDF and SDF were significantly different (*p* < 0.05). The O-SDF treatment resulted in higher levels of IDF (46.17 g/100 g) but less SDF (7.27 g/100 g). By contrast, the S-SDF group showed a significantly reduced content of IDF (22.81 g/100 g) but an increased content of SDF (30.56 g/100 g). This suggests that superfine grinding can convert the IDF into SDF, therefore increasing the utilization of dietary fiber and avoiding the waste of resources. Subsequently, UV spectroscopy was used to detect the distribution of impurities such as proteins in the SDF before and after superfine grinding (Figure 4D). The results showed that the SDF before and after superfine grinding, subjected to extraction by the three-stage enzymatic method, did not show a peak at 280 nm, indicating that both samples contained fewer proteins. This means that the dietary fibers resulting from the O-SDF and S-SDF treatments possess high purity, which allows their subsequent structural characterization and activity verification.

### 3.4 Analysis of the structures

#### 3.4.1 SEM analysis

**Figure 3:**
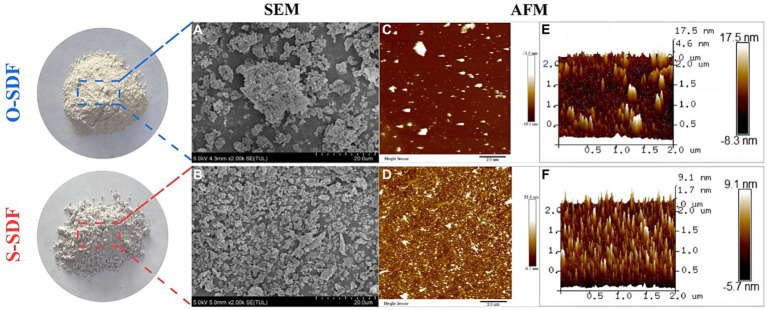
SEM **(A,B)** and AFM **(C–F)** observations of SDF samples.

To compare the differences in morphology before and after superfine grinding (O-SDF and S-SDF), we characterized the appearance of the samples using SEM. O-SDF showed irregular agglomerates of different sizes and had a larger average particle diameter with poor homogeneity (Figure 3A). By contrast, the fragmentation of S-SDF increased after superfine grinding, and the surface structure of the particles was loose and porous, with a smaller average particle diameter and more homogeneous dispersion (Figure 3B). This result was attributed to the strong mechanical shear and cavitation effects of superfine grinding (18). The structure of the dietary fiber was severely damaged by the grinding action, which led to the weakening of the chemical bonds (hydrogen bonding, covalent bonding, and intermolecular bonding) between the dietary fiber molecules (19). These structural changes are more conducive to the conversion of IDF to SDF, which is consistent with the results in Table 5.

**Figure 4:**
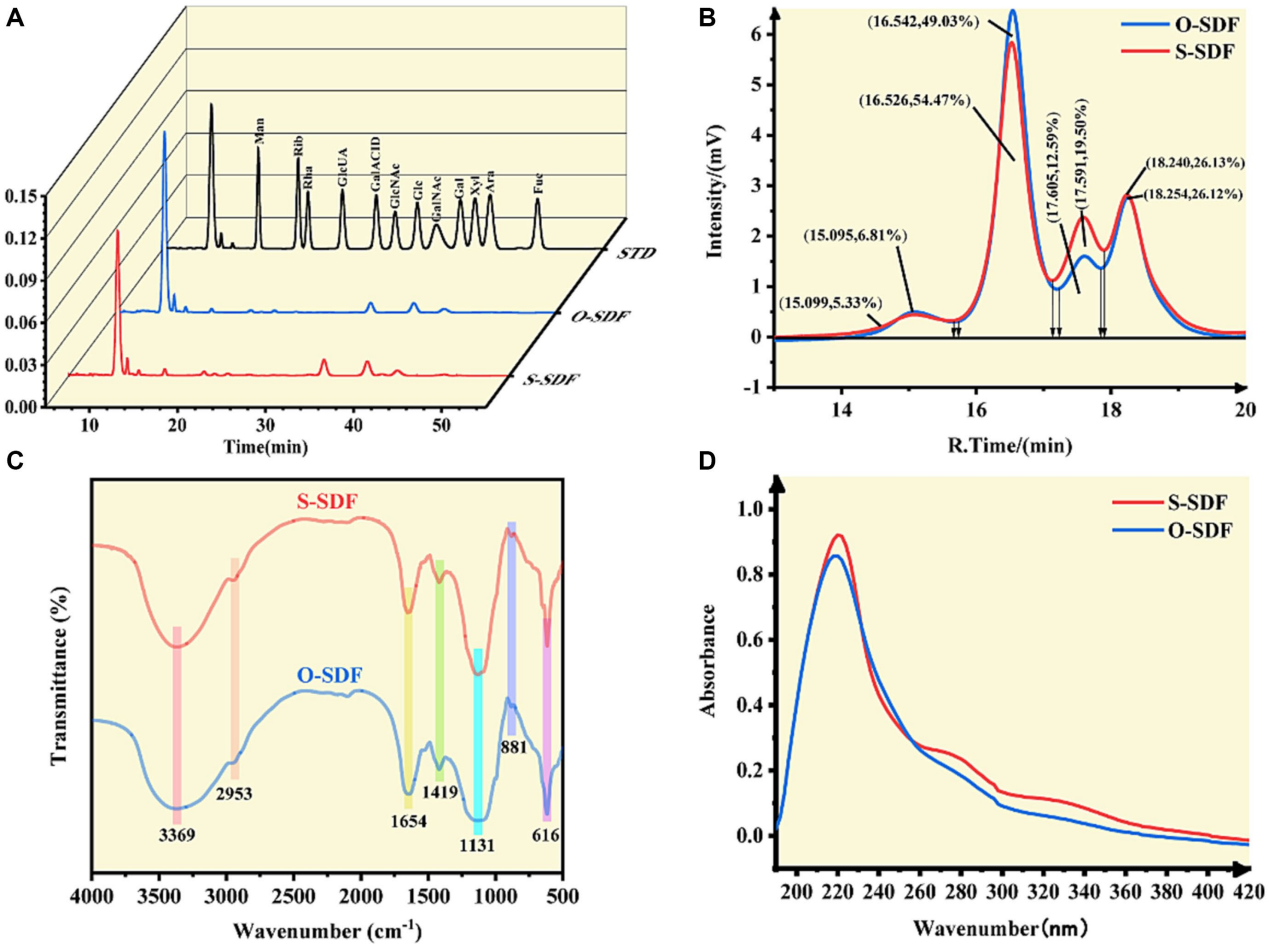
(A) Monosaccharide composition; (B) molecular weight; (C) FT-IR spectra; (D) UV-Vis spectra of SDF samples.

**Table 5:** Composition of the dietary fiber in the tiger nut residues.

Pretreatment method	SDF (g/100 g)	IDF (g/100 g)	TDF (g/100 g)
Ordinary crushing	7.27 ± 0.04^b^	46.17 ± 0.16^a^	53.44 ± 0.19^a^
Superfine grinding	30.56 ± 0.58^a^	22.81 ± 0.21^b^	53.37 ± 0.13^a^

SDF, soluble dietary fiber; IDF, insoluble dietary fiber; TDF, total dietary fiber. The contents are expressed as means ± standard deviation (*n* = 3). Different letters denote significant differences in the same column (*p* < 0.05).

#### 3.4.2 AFM analysis

AFM is a visual scanning probe microscope used to characterize the surface morphology, particle state, and distribution of samples. Figures 3C,D show the molecular surface morphology of O-SDF and S-SDF (scanning range of 10 × 10 μm), respectively. The surface of O-SDF exhibited large loose lamellae and strip-like structures. By contrast, the surface of S-SDF showed tiny and densely structured particles, as well as a few compact lamellar aggregates. Figures 3E,F show the three-dimensional structures of O-SDF and S-SDF in the solution-mica sheet system, respectively. The surface morphology of O-SDF was rugged and exhibited many irregular continuous rod-like protrusions with sharp ends and a maximum aggregation height of 13 nm, whereas S-SDF exhibited dispersed, homogeneous, independent, and regular needle-like protrusions with a maximum aggregation height of 80 nm. This result is consistent with those of SEM, indicating that superfine grinding can destroy the structure of the dietary fibers. Superfine grinding transforms the large molecules of IDF, which have a loose and irregular structure, into SDF with a smaller molecular weight and regular structure (20). These results were comparable to the findings in the literature that superfine grinding changes the molecular weight, intermolecular distance, and interconnection of polysaccharides (21).

#### 3.4.3 Analysis of monosaccharide composition

To compare the variations in the constituents of the SDF molecules before and after modification, we examined their monosaccharide composition using HPLC. The SDF of tiger nut residues was an acidic heteropolysaccharide consisting of glucuronic acid, galacturonic acid, mannose, ribose, rhamnose, glucose, galactose, xylose, arabinose, and fucose (Figure 4A). There was no substantial change in the composition of monosaccharides of the O-SDF and S-SDF. However, the molar percentages in SDF were significantly (*p* < 0.05) increased after superfine grinding (S-SDF): from 1.86 to 2.36% for rhamnose, from 1.54 to 2.40% for galacturonic acid, from 0.7 to 3.24% for fucose, from 6.61 to 7.62% for mannose, and from 1.8 to 3.39% for xylose. These observations are slightly different from previous studies. For instance, Huang et al. suggested that superfine grinding increased the proportion of monosaccharides that make up hemicellulose, including xylose and mannose (13). However, arabinose and galactose decreased significantly (*p* < 0.05). As reported in numerous studies, galacturonic acid and rhamnose are the major constituents commonly found in pectin, and xylose and mannose are typical ingredients of hemicellulose (22). The increase of rhamnose, galacturonic acid, mannose, and xylose in our research indicated that superfine grinding promotes the conversion of IDF to SDF by increasing the content of pectin and hemicellulose. This is consistent with the observed increase in the content of SDF and the decrease in IDF after the superfine grinding treatment.

#### 3.4.4 Analysis of molecular weight

Numerous studies have demonstrated that superfine grinding is effective in altering the molecular structures of target compounds and reducing their molecular weights (23). Therefore, this study compared the molecular weight distribution of O-SDF and S-SDF. The SDF from tiger nut residues exhibited four peaks (Figure 4B) with different molecular weights (Table 6). Compared with O-SDF, the molecular weight of S-SDF decreased from 2.63 × 10^3^ to 1.73 × 10^3^ (Table 6) at 16.53 min, and the peak area decreased from 54.47 to 49.03% (Figure 4B). By contrast, the molecular weight of the S-SDF increased from 4.68 × 10^2^ to 4.98 × 10^2^ at 17.59 min, and the peak area increased from 12.59 to 19.50%. However, the molecular weights of the fractions that showed peaks at 15 and 18 min did not change significantly (Figure 4B). This suggests that superfine grinding can reduce molecular weight and thus increase the content of SDF, which is consistent with previous studies (19). For instance, Maina et al., and Yan et al., suggested that superfine grinding leads to the breakage of the intermolecular chemical bonds between the SDF molecules by decreasing the particle size, resulting in a decrease in the molecular weight and altering the ratio of monosaccharides in the SDF (24). Moreover, the reduction in molecular weight in this research also validated the SEM results.

**Table 6:** Monosaccharide composition and molecular weight of the SDF samples.

	O-SDF		S-SDF	
Monosaccharide composition	Ratio	Mole %	Ratio	Mole %
Mannose	1.00 ± 0.03^a^	6.61 ± 0.16^a^	1.64 ± 0.09^b^	7.62 ± 0.47^b^
Ribose	0.68 ± 0.08^a^	5.58 ± 0.33^a^	1.04 ± 0.11^a^	4.04 ± 0.09^a^
Rhamnose	0.37 ± 0.03^a^	1.86 ± 0.23^a^	0.75 ± 0.09^b^	2.36 ± 0.43^b^
Glucuronic acid	0.37 ± 0.08^a^	1.94 ± 0.29^a^	0.36 ± 0.12^a^	1.92 ± 0.15^a^
Galacturonic acid	0.10 ± 0.04^a^	1.54 ± 0.12^a^	0.15 ± 0.09^b^	2.40 ± 0.84^b^
Glucose	5.33 ± 0.06^a^	29.86 ± 0.17^a^	8.34 ± 0.14^a^	28.24 ± 0.34^a^
Galactose	4.82 ± 0.04^a^	31.08 ± 0.63^a^	6.84 ± 0.05^a^	31.22 ± 0.42^a^
Xylose	0.14 ± 0.05^a^	1.80 ± 0.74^a^	0.58 ± 0.09^b^	3.39 ± 0.59^b^
Arabinose	1.91 ± 0.70^a^	19.03 ± 0.27^a^	2.37 ± 1.01^a^	15.57 ± 0.79^a^
Fucose	0.10 ± 0.11^a^	0.70 ± 0.45^a^	0.70 ± 0.16^b^	3.24 ± 0.27^b^
Molecular weight	O-SDF		S-SDF	
	M_w_ (Da)		M_w_ (Da)	
Fraction 1	3.0601 × 10^4^ ± 45.09^a^		3.2143 × 10^4^ ± 22.03^a^	
Fraction 2	2.632 × 10^3^ ± 15.71^a^		1.734 × 10^3^ ± 8.08^b^	
Fraction 3	4.68 × 10^2^ ± 5.69^a^		4.98 × 10^2^ ± 5.77^b^	
Fraction 4	1.46 × 10^2^ ± 7.57^a^		1.43 × 10^2^ ± 4.58^a^	
M_w_/M_n_				
Fraction 1	1.38 ± 0.03^a^		1.39 ± 0.06^a^	
Fraction 2	1.23 ± 0.06^a^		1.22 ± 0.05^b^	
Fraction 3	1.09 ± 0.03^a^		1.11 ± 0.03^b^	
Fraction 4	1.29 ± 0.04^a^		1.29 ± 0.03^a^	

O-SDF and S-SDF were extracted from SDF by ordinary crushing and superfine grinding, respectively. The ratio represents the amounts of different monosaccharides in different samples compared to the amount of mannose in the O-SDF sample. The mole % is the relative mole % of different monosaccharides in the same sample. Different letters denote significant differences in the same column (*p* < 0.05).

#### 3.4.5 FT-IR analysis

FT-IR spectroscopy was used to characterize the changes in the functional groups of the SDF before and after superfine grinding. Figure 4C shows that both the O-SDF and S-SDF exhibited the typical absorption peaks of carbohydrates, suggesting that superfine grinding did not change the primary structure of the SDF, including the O-H stretching vibration generated by galacturonic acid, xylose, mannose, galactose and arabinose (peak at 3369 cm^−1^). The absorption peaks at 2953 cm^−1^ [methyl and methylene (C-H) stretching of carbohydrates] and 1,131 cm^−1^ (C-O, C-C, and C-O-C stretching, which is usually found in arabinoxylan and xylan) were also retained (19). The absorption peak at 881 cm^−1^ corresponds to the β-type pyranose ring, and 616 cm^−1^ indicates the vibrational absorption peak of the β-type C-H bond in the carbohydrate molecule (25–27). In addition, the absorption peak at 1654 cm^−1^ corresponds to the aromatic skeleton and C=O stretching in aldehydes or esters. The weak absorption peaks near 1,654 cm^−1^ and 1,419 cm^−1^ represent the ester carbonyl group (C=O) and the carboxyl group (-COOH), suggesting that uronic acid is present in the SDF (14). Notably, the peaks at 1654 cm^−1^, 1,419 cm^−1^, and 1,131 cm^−1^ are considered to be the absorption peaks of low-methyl pectin and are consistent with the results of the monosaccharide composition analysis.

### 3.5 Cation-exchange capacity

**Figure 5:**
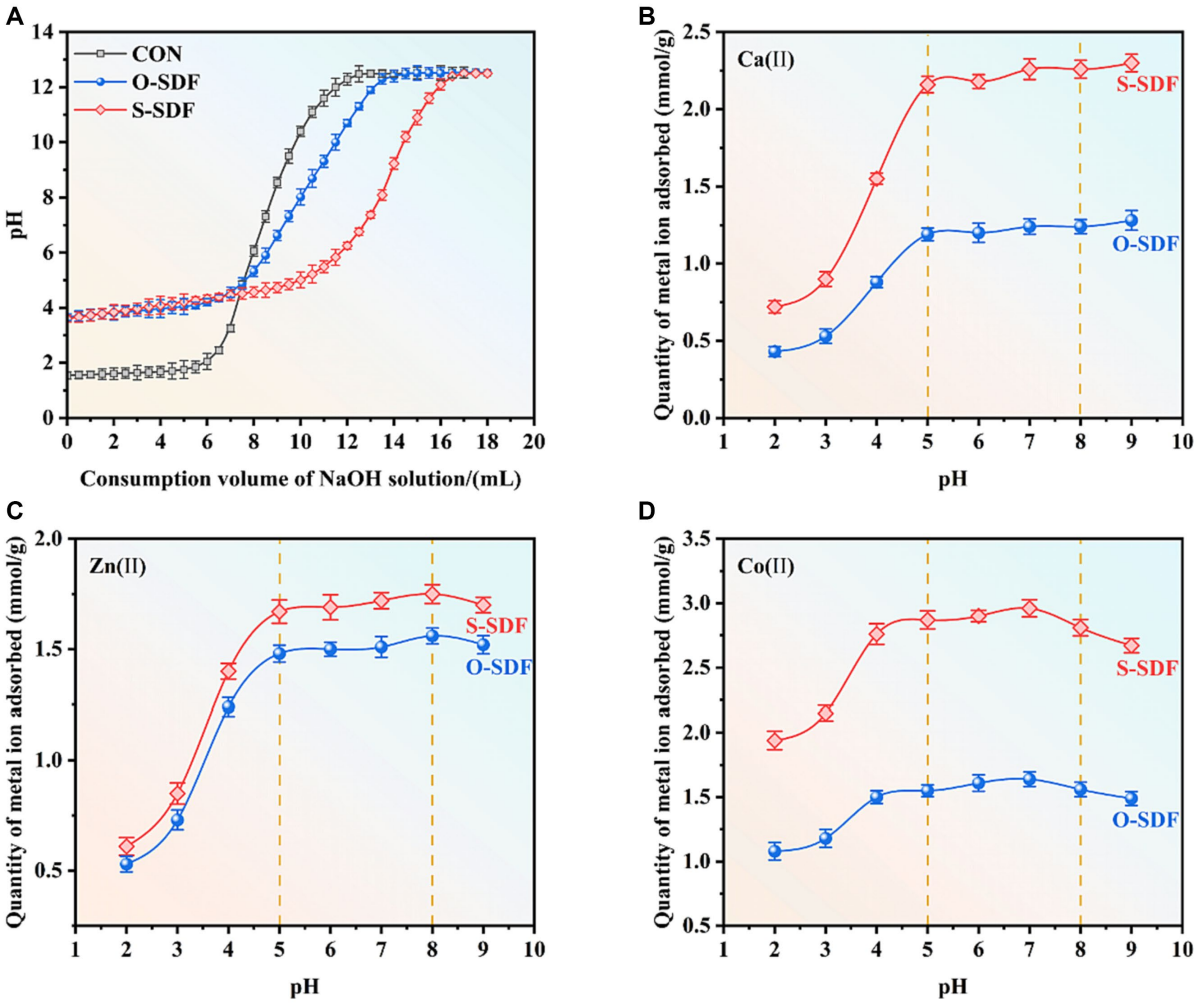
**(A)** Cation-exchange capacity; **(B–D)** metal-ion-binding properties of SDF samples.

Research has shown that SDF contains numerous reactive groups such as carboxyl and hydroxyl groups, which can effectively adsorb and exchange cations (28). To investigate the ion-exchange capacity of SDF before and after superfine grinding, we acidified SDF using hydrochloric acid to dissociate H^+^ from the carboxyl and hydroxyl groups. Subsequently, a NaOH solution was added to neutralize H^+^ after the protonation of SDF. The stronger the cation adsorption capacity of SDF, the more H^+^ would be incorporated during the acidification process. Therefore, when the pH stabilized, the system required more NaOH. The pH of the control group increased considerably and showed the maximum slope as the NaOH solution was added. By contrast, the pH of the O-SDF and S-SDF increased steadily Figure 5A. This suggests that the SDF from tiger nut residues has a high cation-exchange capacity. Furthermore, the cation-exchange capacity of the S-SDF was substantially higher than that of the O-SDF, which suggests that superfine grinding decreased the particle size of the SDF and exposed the active groups (e.g., carboxyl and hydroxyl), thus resulting in a higher cation-exchange capacity.

### 3.6 Metal-ion-binding properties

Previous studies have shown that dietary fibers have desirable ion-exchange and cation adsorption capacities (29). Therefore, we chose several essential elements (Ca^2+^, Zn^2+^, and Co^2+^) to evaluate the ion adsorption effect of SDF from tiger nut residues after superfine grinding (Figures 5B–D). S-SDF exhibited a higher metal-binding capacity (Ca^2+^, Zn^2+^, and Co^2+^) than O-SDF. The adsorption curves of both S-SDF and O-SDF for the metal ions in the reaction system increased as the pH increased, followed by a slight weakening after the adsorption stabilized. This is likely due to the fact that superfine grinding exposes a greater number of functional groups such as free carboxyl or hydroxyl groups (which are negatively charged) (30), and these reactive groups (carboxyl and hydroxyl) are protonated negatively in acidic environments with pH values of 2–5 (14) via interact with metal cations by electrostatic adsorption (31). To maintain the binding sites deprotonated and maximize binding to the metal ions, we used OH-to consume these conjugated H^+^, thereby promoting the substitution of metal ions for H^+^ in the SDF to achieve adsorption equilibrium. This adsorption equilibrium is interrupted at pH ranging from 5 to 8, which results in a slight reduction of the adsorption capacity. These findings are consistent with other research indicating slightly acidic conditions are more favorable for the adsorption of metal ions, while adsorption decreases slightly under weakly alkaline conditions due to the formation of insoluble metal hydroxides in this condition (32).

### 3.7 Antioxidant capacity analysis *in vitro*

**Figure 6:**
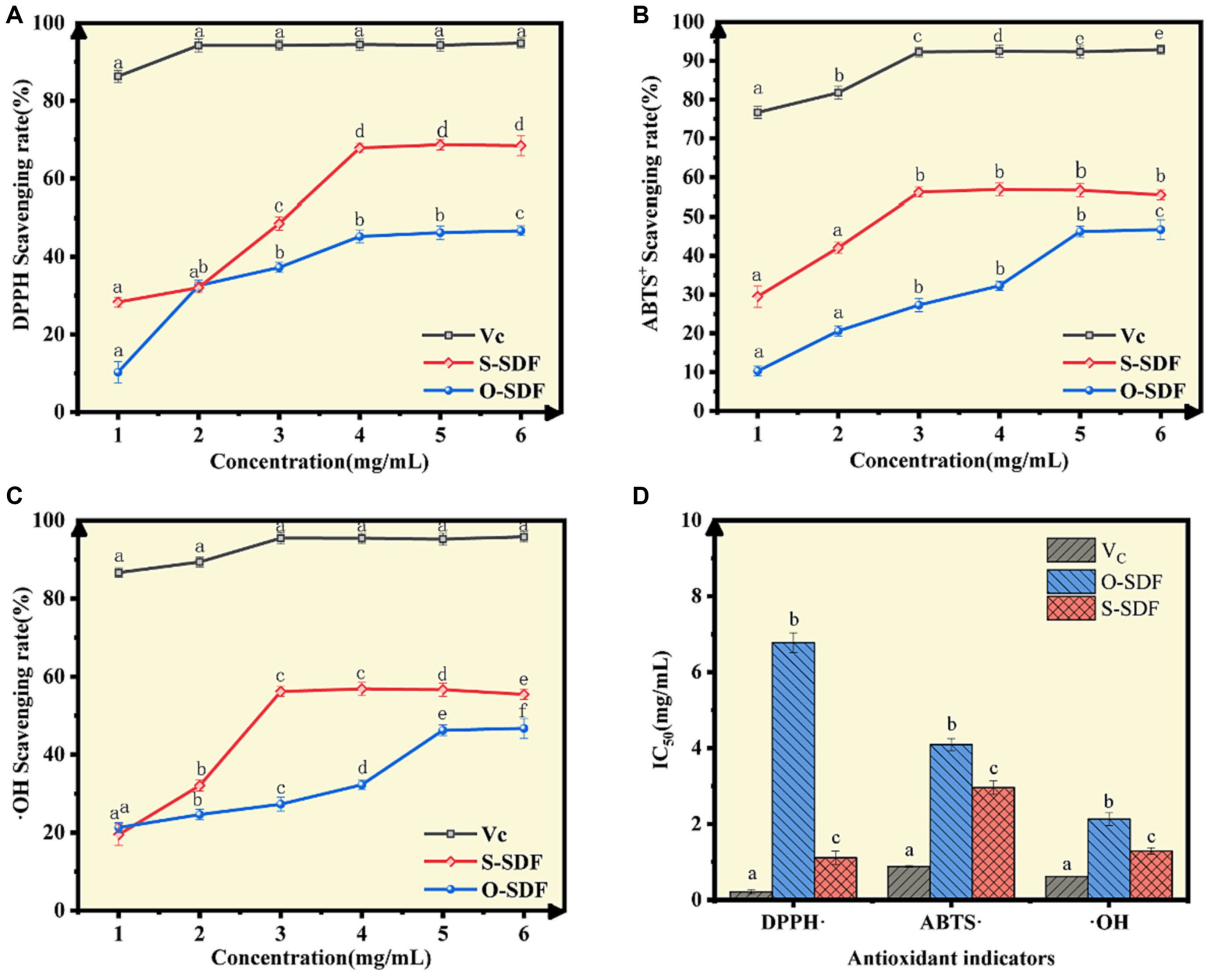
Antioxidant capacity of SDF samples *in vitro*. **(A)** DPPH radical-scavenging activity; **(B)** ABTS radical-scavenging activity; **(C)** Hydroxyl radical-scavenging activity; **(D)** Half-maximal inhibitory concentrations (IC_50_) of several antioxidants.

We examined the antioxidant properties of the SDF before and after superfine grinding. The antioxidant capacities of O-SDF and S-SDF against three free radicals (ABTS^+^, DPPḤ, and ·OH) increased in a dose-dependent manner (concentrations from 1 mg/mL to 6 mg/mL), and the antioxidant capacity of S-SDF was significantly higher than that of O-SDF (*p* < 0.05; Figures 6A–C). S-SDF achieved the maximum DPPH-removal (68.66%) at a concentration of 5 mg/mL, with an IC_50_ value of 1.11. However, O-SDF achieved the maximum removal (46.72%) at a concentration of 6 mg/mL, with an IC_50_ value of 6.78 (Figures 6A,D). In addition, S-SDF achieved the maximum removal of ·OH at 56.84% with an IC50 value of 1.29, and it also exhibited the highest removal of ABTS^+^ at 46.24% with an IC50 value of 4.09, both observed at a concentration of 3 mg/mL (Figures 6B–D). By contrast, O-SDF achieved the maximum clearance of ·OH (46.72% with an IC_50_ value of 2.13) and ABTS^+^ (46.24% with an IC_50_ value of 4.09) at a concentration of 5 mg/mL; Figures 6B–D). This result demonstrates that superfine grinding enhances the antioxidant capacity of the SDF from tiger nut residues. Indeed, the improved antioxidant capacity of dietary fibers after superfine grinding contributes to several aspects, including the reduction in molecular weight, increase in specific surface area, and higher proportion of acidic heteropolysaccharides (uronic acid contents). Numerous studies have confirmed those findings that lower molecular weights, and acidic heteropolysaccharides containing galacturonic acid, uronic acid, etc. usually exhibit substantially higher antioxidant capacity (21, 33, 34).

## 4 Conclusion

Our findings suggest that the molecular structure of the soluble dietary fiber from tiger nut residues before and after superfine grinding was disrupted under the strong mechanical shear of this method, resulting in a lowered molecular weight and increased proportion of functional monosaccharides, as well as an improvement in the ion adsorption capacity and antioxidant activity. Therefore, dietary fibers can be modified using superfine grinding to improve their functionality. Importantly, such superfine grinding technology also promotes efficient utilization of tiger nut by-products and provides data to support the development of functional fiber-rich nutritional supplements.

## Data availability statement

The original contributions presented in the study are included in the article/supplementary material, further inquiries can be directed to the corresponding authors.

## Author contributions

WW: Writing – original draft, Writing – review & editing. ZQ: Writing – review & editing. FK: Writing – original draft, Writing – review & editing. SZ: Writing – review & editing. LC: Writing – review & editing. ZZ: Writing – review & editing.
